# Outcomes of Knee Dislocations and Subluxations in Polytrauma Patients at a Major Trauma Center

**DOI:** 10.7759/cureus.75024

**Published:** 2024-12-03

**Authors:** Benjamin Gompels, David Holdroyd, Jonathan Bennett, Estelle Strangmark, Tom Molloy, Reece Barter, Simone Castagno, Stephen McDonnell, Jai Rawal

**Affiliations:** 1 Department of Trauma and Orthopaedics, Addenbrooke's Hospital, Cambridge University Hospitals NHS Foundation Trust, Cambridge, GBR; 2 Department of Surgery, University of Cambridge, Cambridge, GBR; 3 Department of Trauma and Orthopaedics, University of Cambridge, Cambridge, GBR; 4 Department of Trauma and Orthopaedics, Imperial College London, London, GBR

**Keywords:** knee dislocation, knee injuries, knee trauma, multiple trauma, polytrauma, traumatic knee dislocation

## Abstract

Background

This is a retrospective service evaluation of outcomes of polytrauma patients sustaining knee dislocations and subluxations within a major trauma center (MTC). Polytrauma patients with knee dislocations are complex to manage and often sustain multiple life-threatening injuries. Although treatments have progressed, no consensus remains on management timing and strategy.

Methods

Information on knee dislocations and subluxations sustained in polytrauma patients was extracted using the Trauma Audit and Research Network database. This included demographic information on age, gender, and BMI, as well as information regarding their initial assessment in the emergency department (ED), including the Glasgow Coma Scale, Injury Severity Score, mechanism of injury, and time spent in ED. Associated injuries to the knee joint, including vascular compromise, associated bony injury, and ligamentous damage, were extracted from the patient notes. The management of these injuries was also examined, including the operative time, whether they required single or multistage surgery, and post-operative range of movement.

Results

Over eight years, from 2013 to 2021, there were a total of 187 polytrauma patients sustaining knee injuries, of which 17 had a knee joint dislocation and seven had a knee joint subluxation. The mean age of the patients was 46, their BMI was 28, and 19 out of the 24 had been involved in a vehicle incident or collision. In 80% of patients, there was an associated fracture of the lower limb, and just under half of patients had associated ligamentous injury (11 patients), with one-third of the patients sustaining an associated vascular injury (eight patients). One-third of the patient cohort was managed conservatively in a brace. Of the remaining, 12 required operative fixation, three underwent an amputation, and one was managed non-operatively with plaster of Paris. The mean operative time was 80 minutes in patients with vascular injury compared to 159 minutes without. This could be attributed to the more complex injuries in polytrauma that accompany a vascular injury. The median operative time was 71 minutes and 80 minutes, respectively. Within this study, the distance to the MTC did not appear to be a key determinant of amputation, though the low patient numbers limit the statistical power of this study.

Conclusions

Polytrauma patients are a complex cohort, often sustaining multiple injuries that require operative management with a multidisciplinary approach. Knee dislocations are a rare injury and are high energy, primarily caused by vehicle collisions in a polytrauma setting. A third of patients have an associated vascular injury, and 80% have an associated fracture, which is a much higher rate than is observed in a non-polytrauma setting.

## Introduction

Polytrauma patients exhibit a minimum of two severe injuries in one or more areas of the body. These cases can stem from high-energy trauma incidents, such as motor vehicle accidents, falls, industrial mishaps, and sports-related injuries, or even lower-energy incidents like stepping off a curb [1-3]. Given the concurrence of multiple injuries, primary surveys can often overlook lower limb injuries, resulting in delayed treatment and exacerbated functional long-term outcomes [4].

Among polytrauma cases, tibiofemoral dislocations, also known as knee dislocations, represent a rare yet severe form of lower limb injury. They disrupt many or all of the four major ligaments stabilizing the knee joint and demand urgent surgical attention due to the potential for limb-threatening complications, notably vascular injury. Current literature, however, needs more consistency on the prevalence of concomitant vascular injury in knee dislocation cases. The popliteal artery, which spans the popliteal space and is the primary blood supply to the distal lower extremity, is especially vulnerable to injury in knee dislocations. As such, timely assessment of vascular status through distal and popliteal pulse palpation, ankle-brachial pressure index measurement, arterial duplex ultrasound, and, when indicated, CT angiography is pivotal in averting lower limb ischemia, necrosis, and amputation [5,6]. In the context of polytrauma, this process is complicated by the simultaneous presence of multiple life-threatening injuries necessitating thorough investigation and treatment.

This study evaluates the prevalence and outcome of knee dislocations or subluxations among the 187 high-energy polytrauma patients sustaining knee injuries reported in the Trauma Audit and Research Network (TARN) database from 2013 to 2021. We aim to highlight the rates of associated injuries, such as vascular or ligamentous damage, and provide insight into managing these lower limb injuries at a major trauma center (MTC).

## Materials and methods

This study was registered as a service evaluation at Addenbrookes Hospital, Cambridge University Hospitals NHS Foundation Trust, a tertiary center in the East of England that is an MTC for the region. Using the TARN database, information on knee dislocations and subluxations sustained by polytrauma patients was extracted over eight years from 2013 to 2021. This consisted of demographic information on age, gender, and body mass index (BMI), as well as information regarding their initial assessment in the emergency department (ED), including Glasgow Coma Score (GCS), Injury Severity Score (ISS), mechanism of injury, and time spent in ED. The associated injuries to the knee joint, including vascular compromise, associated bony injury, and ligamentous damage, were extracted from the patient notes. The management of these injuries was also examined, including whether they required single or multistage surgery, operative time, and postoperative range of movement from the outpatient follow-up clinic. Additionally reviewing patients' notes, the pre-hospital ambulance sheet was used to calculate the miles from the incident to the MTC and the time from incident to surgical intervention for patients with and without vascular injuries. Basic statistical analysis of this was performed on the distance to MTC and operative time using the Wilcoxon rank test.

Statistical analysis was also conducted on the operative time and time spent in the ED for patients who sustained vascular injuries and patients who did not. This used a Wilcoxon rank test to look for differences between the two groups. An odds ratio for the likelihood of patients sustaining a vascular or ligamentous injury based on whether they suffered a knee dislocation or subluxation was also performed. All statistical analyses were performed on GraphPad Prism version 9.5.0 (525) 2022 (Dotmatics, Boston, Massachusetts). Results were considered statistically significant if the p-value was less than 0.05.

## Results

Overall injuries

Over eight years, from 2013 to 2021, there were a total of 187 polytrauma patients sustaining knee injuries, of which 17 had a knee joint dislocation and seven had a knee joint subluxation.

Patient demographic

The mean age of the patients sustaining knee dislocations or subluxations as a result of polytrauma was 46 years (range 16-90), and the mean BMI was 28. Most patients were male (18) compared to female (6).

Admission

The mean GCS of patients arriving at the trauma center was 13.5 (range 3-15), and the ISS on arrival in the ED was 23. The most bodily region was limb injury. The mean length of stay in the hospital for this cohort of patients was 774 hours (approximately 32 days), and the mean length in the intensive care unit was 130 hours (about five days). At 30 days, 22 of the 24 total patients were still alive, with two mortalities. In 18 of the 24 patients, an entire trauma team was present on arrival in the ED. Approximately 30% of the patient cohort was in systolic shock (defined as systolic blood pressure of <110 mmHg) on arrival in the ED.

Mechanism of injury

Nineteen of the 24 had been involved in a road traffic collision. Of the remaining five patients, four sustained falls, and one was involved in a blow without a weapon.

Injury type

**Figure 1:**
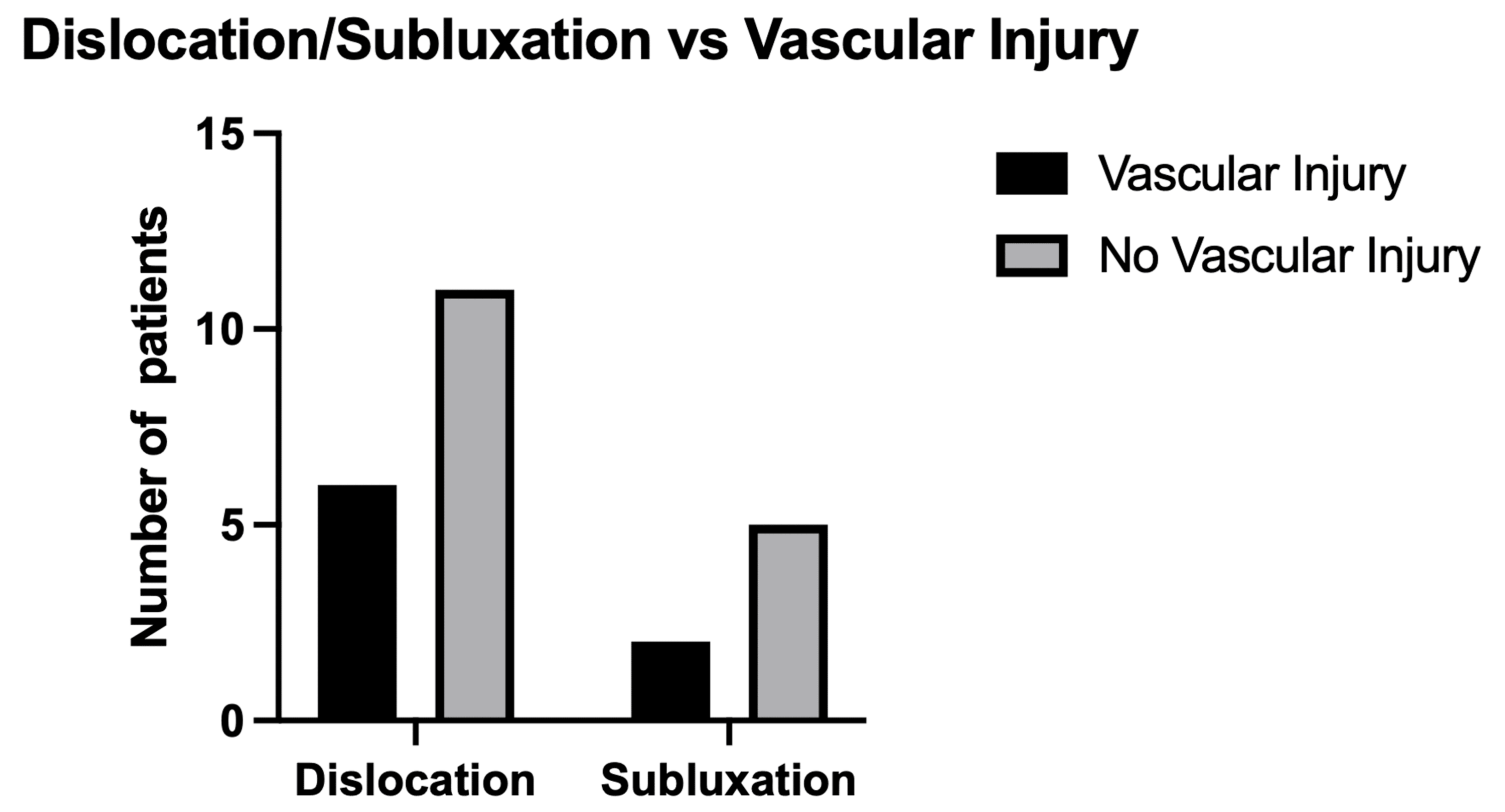
Number of patients sustaining a dislocation or subluxation with a concurrent vascular injury

Of 24 patients, 17 had a knee joint dislocation and seven had a knee joint subluxation. In 80% of patients, there was an associated lower limb fracture, and associated ligamentous injury was reported in just under half of patients (10). One-third of the patients sustained an associated vascular injury (8 out of 24). Patients who had a knee dislocation were more likely to have a vascular injury than those who had a subluxation (odds ratio: 1.4; 95% CI 0.2-9.2; p>0.999). The number of patients who sustained a vascular injury for dislocation and subluxation is visualized in Figure 1.

**Figure 2:**
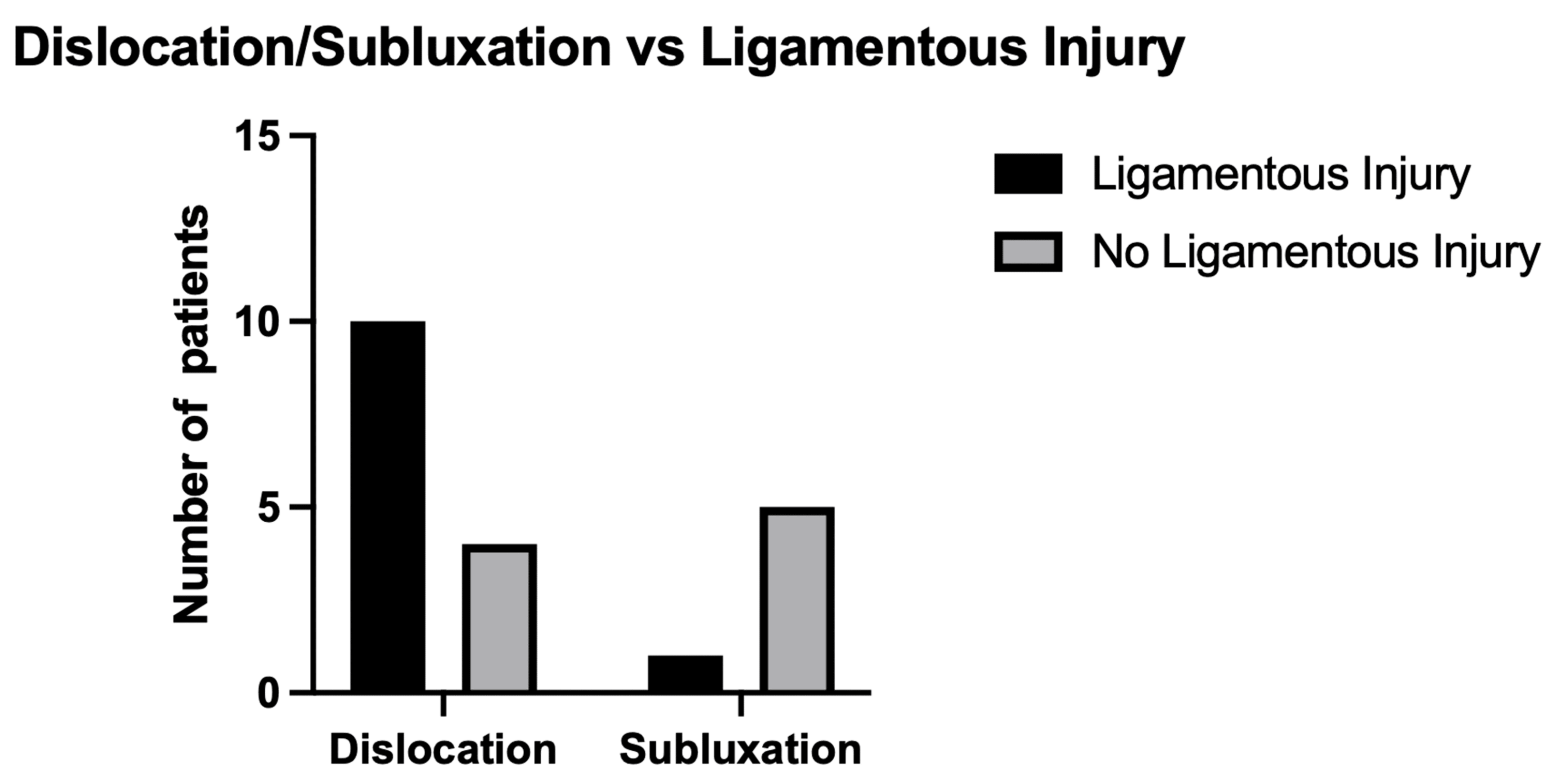
Number of patients sustaining a dislocation or subluxation with a concurrent ligamentous injury

Patients who sustained a dislocation were much more likely to maintain a ligamentous injury (odds ratio: 12.5; 95% CI 2.5-143.3; p=0.0498). The number of patients sustaining a ligamentous injury after having a subluxation or dislocation is shown in Figure 2.

Ligamentous injuries

Eleven patients had ligamentous injuries. The most frequently affected ligaments were the anterior cruciate ligament (ACL) and posterior cruciate ligament (PCL), with 11 and 10 patients sustaining damage to either ligament, respectively. The collateral ligaments were injured less frequently, with seven patients experiencing medial collateral ligament (MCL) injury and four patients with damaged lateral collateral ligament (LCL).

Treatment

Most patients underwent surgery within 24 hours of arriving in the ED (17 out of the 24). The mean operative time was 80 minutes (range 12-174 minutes) in patients with vascular injury compared with 159 minutes (range 10-458 minutes) in patients with no vascular injury (p=0.56). Of the patients who sustained vascular injuries, one patient died, and three patients required an amputation. Three required external fixation and restoration of flow were not patent, and one had an aneurysm that did not require surgical intervention.

Initial management of knee dislocation or subluxation

**Figure 3:**
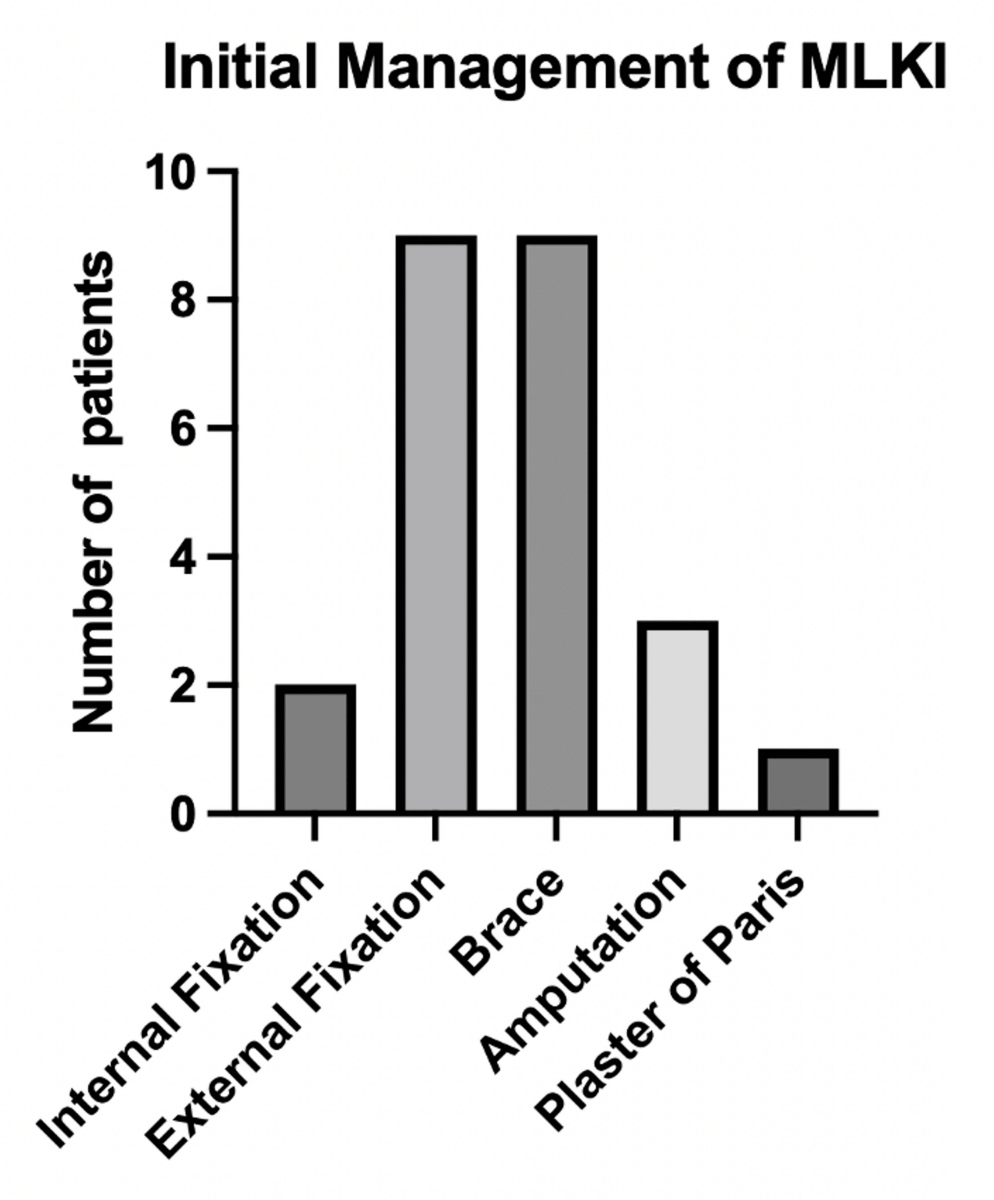
Number of patients who received each management option after sustaining either a dislocation or subluxation MLKI: multi-ligament knee injury

Knee dislocations or subluxations were initially managed in a brace, with operative fixation (internal or external) or amputation. Of 24 patients, nine had a brace fitted initially, two had internal fixation, nine had external fixation, three had amputations, and one was managed in plaster with a back slab. This is visualized in Figure 3.

Minutes from the time of injury to surgical intervention

**Figure 4:**
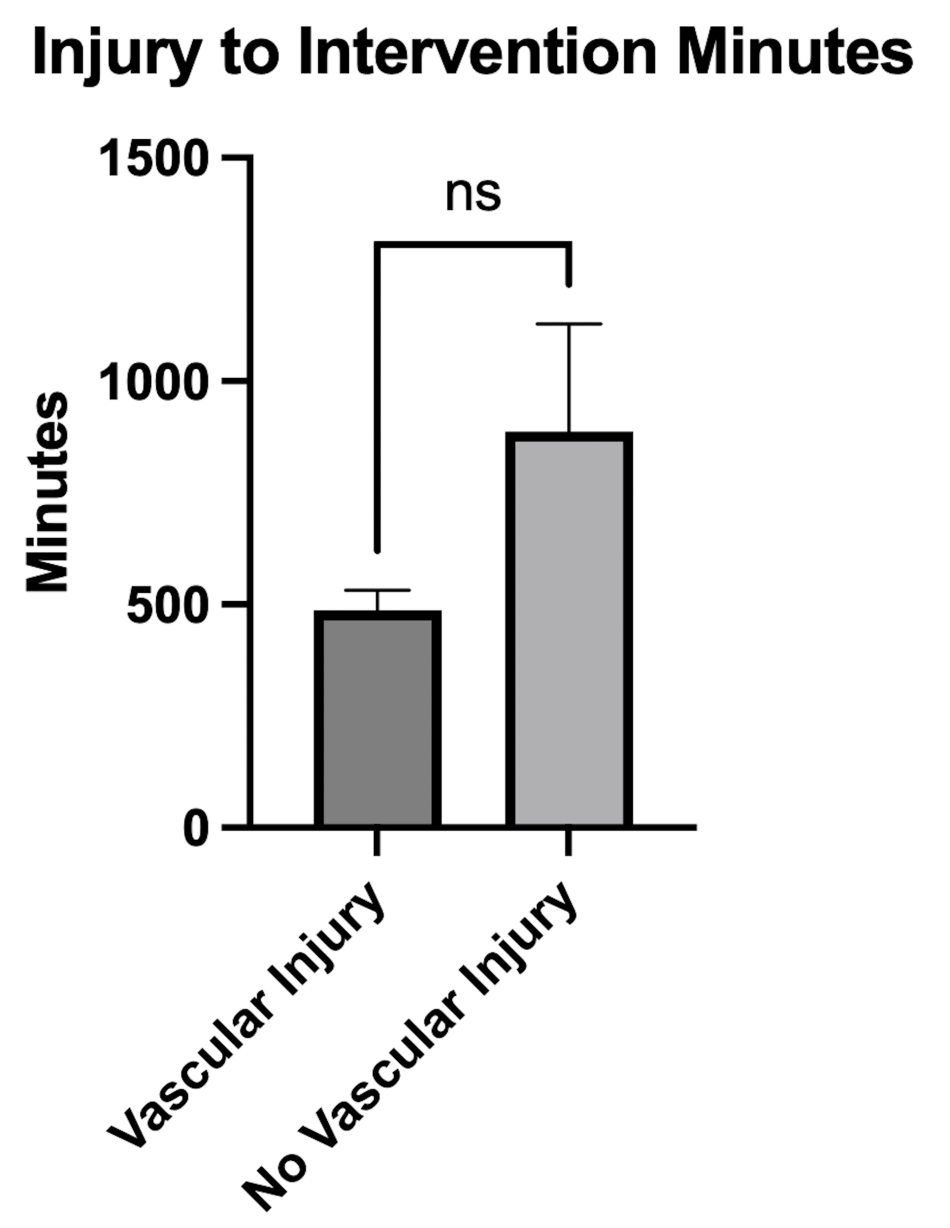
Total time from injury to surgical intervention

Using data from the ambulance records, we calculated the time difference between the time of injury and the commencement of surgical intervention for two groups: those with vascular injuries and those without. The analysis revealed a mean time of 486 minutes (with a range spanning from 351 to 628 minutes) for patients with vascular injuries, while patients without vascular injuries had an average duration of 887 minutes (ranging from 550 to 2212 minutes) (p=0.10). This finding is visually represented in Figure 4.

We also examined the proximity of the incident location to the MTC for individuals who underwent amputation compared to those who did not. A relatively small number of patients (n=3) underwent amputation in our study. Among these patients, the average time from the incident to arrival at the MTC was 521 minutes, with a range spanning from 360 to 628 minutes. The mean duration for those who did not require amputation was 756 minutes, ranging from 239 to 2212 minutes (p=0.81).

Miles from the incident to the major trauma center

Among the 24 patients studied, data was unavailable for a subset (10 patients) due to either missing ambulance records or their admission predating the implementation of the Epic system. For the patients for whom data was accessible, an analysis of the distance in miles from the MTC to the incident location was conducted. Specifically, in the group with no vascular injuries, the mean distance from MTC to the incident location was 38.2 miles (ranging from 22 to 62 miles), whereas in the group that sustained vascular injuries, the mean distance was 28.7 miles (p=0.52).

A limited number of patients (n=3) required amputation in this study. Among this subset, the average distance for those with vascular injuries was 27.6 miles (ranging from 2.7 to 42 miles). Conversely, patients who did not undergo amputation had a mean distance of 36.8 miles (ranging from 16 to 62 miles) (p=0.55). Neither of these results were statistically significant (p>0.05).

## Discussion

Knee dislocations represent a significant challenge within polytrauma care. Despite their relatively infrequent occurrence, estimated to be less than 0.2% of all orthopedic injuries sustained, suspected knee dislocations are potentially limb-threatening and should be treated as a surgical emergency [7]. This study extracted data from the TARN database to examine the outcomes of knee dislocations and subluxations over eight years at an MTC.

This study confirms the patient demographic susceptible to knee injuries and the high level of trauma they face. Most patients were males, with a mean age of 46. Previous studies have similarly shown the younger male demographic to be an at-risk population [8,9]. However, female patients still make up a significant proportion [10]. Patients presenting with knee dislocation suffer a high burden of trauma with a mean ISS of 23. Almost 60% of multiple trauma patients sustain a significant extremity injury, which can be associated with worse outcomes [11].

In this study, there was a high rate of accompanying vascular injury, with almost a third of patients affected. There is a significant variation in the literature regarding the rates of vascular compromise in patients presenting with knee dislocation or subluxation, with one large-scale study of 8,000 patients citing an overall frequency of 3.3%. However, other studies have quoted up to 30% [9,12]. In many studies, such discrepancies in reported rates often can be attributed to the low prevalence of knee dislocations [13]. Anatomically, due to its location in the popliteal space, the popliteal artery emerges as the most susceptible vascular structure [14]. This could be attributed to the high ISS/burden of trauma faced by patients and underscores the importance of thorough assessment and identification of any neurovascular compromise to prevent limb loss.

Knee dislocations are associated more frequently with ligamentous damage. Studies have suggested that, in most cases, damage to at least one cruciate ligament is necessary to dislocate the knee [15]. This is supported to an extent by our data, with 10 dislocations reporting a ligamentous injury. The four instances in which dislocation occurred without reported ligamentous injury may potentially be explained by a lack of investigation and/or imaging to rule out damage. Most commonly, traumatic knee dislocations involve a bicruciate injury, with additional MCL/PCL injuries occurring depending on the mechanism of the dislocation [7]. Our data support this, with 91% of ligamentous injuries involving both ACL and PCL. Concurrent ligament injury is common and should, therefore, always be thoroughly investigated in patients with traumatic knee dislocation.

Many factors affected the time elapsed from injury to surgical intervention. First, there was a difference in the mean time to intervention between patients with vascular injuries and those without. Patients with vascular injuries experienced a mean time of 486 minutes, while patients without vascular injuries had an average duration of 887 minutes. However, this difference did not reach statistical significance (p=0.10), possibly reflecting the study's low statistical power and the effect of other factors on time to intervention.

Delays in the identification and treatment of vascular injury increase the risk of irreversible damage [16]. Despite this, we did not find vascular injuries associated with an increased mean distance from the incident location to the MTC compared with those without vascular injuries (p=0.52). Limited cases were focused on the subset of patients who underwent amputation (n=3). The patients experienced an average of 521 minutes from the incident to arrival at the MTC. Comparatively, those who did not require amputation had a mean duration of 756 minutes. Importantly, statistical analysis did not indicate a statistically significant difference (p=0.81) between these groups regarding the distance covered. A larger cohort would be required to conclude this specific subset accurately. However, as highlighted, this could be difficult due to the relatively low incidence of this type of injury.

Several limitations must be acknowledged. Notably, the retrospective nature of the study, relying on data extracted from the TARN database, might result in potential biases or incomplete information. Additionally, the small sample size within the study may compromise the generalisability of some of its findings. Larger studies over a longer period are needed to confirm the findings of this study.

## Conclusions

Polytrauma patients are a complex cohort, often sustaining multiple injuries that require operative management with a multidisciplinary approach. Knee dislocations are rare injuries, usually high-energy, primarily caused by vehicle collisions. A third of patients in this study had an associated vascular injury, which is a higher rate than that observed in a non-polytrauma setting in the literature. The timing of surgical intervention and the distance to the MTC for patients require a larger dataset to draw more substantial conclusions. Due to the low incidence of these injuries, collaborative studies may provide the patient numbers needed to draw more significant findings. In addition, more substantive patient outcome measures would offer more insight into how effectively these patients are managed.
